# Tracheostomy Timing in Unselected Critically Ill Patients with Prolonged Intubation: A Prospective Cohort Study

**DOI:** 10.3390/jcm13102729

**Published:** 2024-05-07

**Authors:** Pınar Tekin, Azime Bulut

**Affiliations:** Department of Anesthesiology and Reanimation, Faculty of Medicine, Giresun University, 28100 Giresun, Türkiye; pinar.tekin@giresun.edu.tr

**Keywords:** tracheostomy timing, mortality, intensive care, prolonged intubation, weaning

## Abstract

**Background**: Tracheostomy procedures are performed in the intensive care unit (ICU) for prolonged intubation, unsuccessful weaning and infection prevention through either percutaneous or surgical techniques. This study aimed to outline the impact of tracheostomy timing in the ICU on mortality, need for mechanical ventilation, and complications. **Methods**: Patients were included in the study on the day of tracheostomy. Demographic information, tracheostomy timing, technique, complications, sedation requirement and need for mechanical ventilation at discharge were recorded by an anesthesiologist, including the pre-tracheostomy period. **Results**: Tracheostomy was performed on 33 patients during the first 14 days of intubation and on 54 patients on the 15th day and beyond. There was no significant difference between the tracheostomy timing and mortality, sedation requirement, or weaning from the ventilator. We observed that patients who underwent tracheostomy with the surgical technique experienced more complications, but there was no significant difference. Tracheostomy performed after the 14th day was shown to be associated with prolonged hospital stay. **Conclusions**: Early tracheostomy does not have any influence on the need for mechanical ventilation, sedation and mortality. The optimal timing for tracheostomy is still controversial. We are of the opinion that randomized controlled trials involving patient groups with similar survival expectations are needed.

## 1. Introduction

Tracheostomy is often performed in cases of prolonged mechanical ventilation due to failed extubation or when extubation is delayed [1,2,3,4]. Approximately 20–24% of intubated intensive care unit patients undergo tracheostomy [2,5]. While the rate of tracheostomy in general intensive care unit patients range from 10 to 15%, it can increase to around 35% in patients with intubation after neurological events [6]. The timing of tracheostomy is influenced by the severity of the disease, the clinician’s preference, the patient and family expectations, the hospital resources, and the expected survival. The optimal timing for performing tracheostomy after a prolonged intubation, and whether early tracheostomy has a positive effect on mortality, the length of hospital stay, and complications, is still a subject of debate. Some studies classify early tracheostomy as that performed within the first 7 days for the prolonged intubation, while others consider those performed within the first 10 or 14 days as well [3,4,7]. The positive effect of early tracheostomy (ET) on the mortality and length of hospital stay has been studied in both specific patient groups and unselected ICU patients; however, no definitive conclusion has been reached. The superiority of early tracheostomy over late tracheostomy is still controversial, and there are limited data to guide the ideal timing for a tracheostomy. Although the exact definition of early tracheostomy is debated, there is greater consensus that late tracheostomies include those performed after 14 days.

In addition to the timing, the selection of technique can also vary depending on the patient and the physician. Both techniques have their advantages and disadvantages; however, anesthesiologists tend to prefer the percutaneous technique, while Ear–Nose–Throat specialists prefer the surgical technique. Percutaneous tracheostomy is a safe procedure that can be performed at the bedside with a low complication rate [8,9,10,11]. The reason for the high complication rates in surgical tracheostomy may be the avoidance of percutaneous tracheostomy in patients with challenging situations such as difficult anatomy and bleeding dysfunction. Early and late complications after tracheostomy include pneumothorax, bleeding, subcutaneous emphysema, wound infection, tracheal stenosis, esophageal injury and tracheoesophageal fistula [9].

Tracheostomy performed due to prolonged endotracheal intubation aims to reduce the need for sedation, enhance patient mobility, provide a safer airway, increase patient comfort, facilitate airway cleaning with aspiration, allow for early discharge from intensive care, reduce direct endolaryngeal damage, enable oral feeding, speech, and communication, decrease airway resistance, and reduce the risk of nosocomial pneumonia [11,12,13,14]. As the duration of mechanical ventilation increases, ventilator-associated pneumonia is likely to increase, leading to a longer length of hospital stay and higher mortality. Therefore, shortening the intubation period with tracheostomy has a positive effect on mortality.

Herein, we aimed to investigate the difference in the outcome rates of patients who underwent tracheostomy within 14 days compared to more than 14 days after intubation. The primary endpoints of the study were hospital mortality and ICU mortality. The secondary endpoints were the length of the ICU stay, the sedation need, being ventilator free, and the tracheostomy complications.

The main research questions of the study were as follows:

- Does performing tracheostomy before the 14th day due to prolonged intubation improve the patient prognosis?
- Is there a difference in the sedation requirement, ICU length of stay and mortality with the timing of tracheostomy?
- Is there a difference in complications between tracheostomized ICU patients based on technique, whether percutaneous or surgical?
- Is there a difference between the successful decannulation and being ventilator-free between tracheostomized ICU patients based on timing?

## 2. Materials and Methods

### 2.1. Study Design

This was an observational, prospective study conducted in a general 24-bed ICU coordinated by the Department of Anesthesiology and Reanimation in a tertiary hospital. This study was approved by the local ethics committee (Giresun University, KAEK-41, 21 April 2020). Informed consent was obtained for all subjects involved in the study.

### 2.2. Study Population

**Figure 1:**
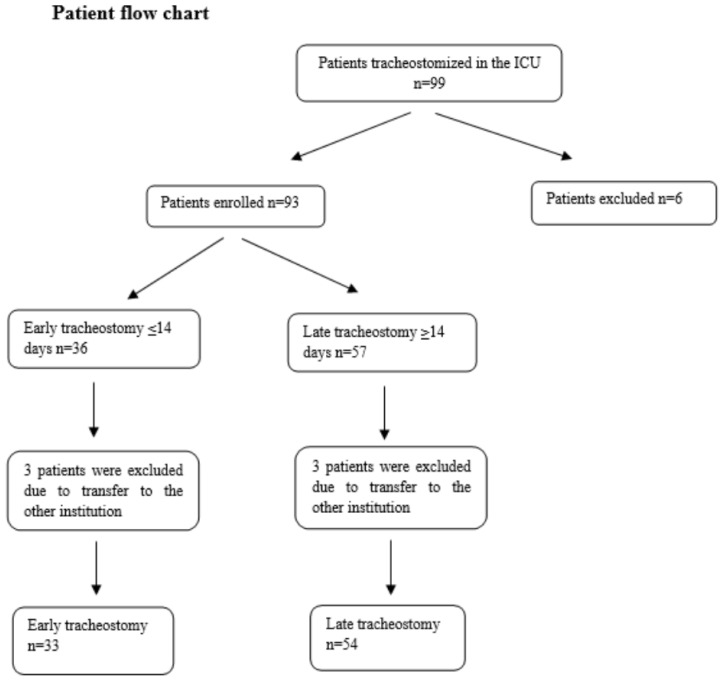
Patient flow chart. ICU: Intensive care unit; *n*: number.

G*Power (V3.1) software (Informer Technologies, Inc., Los Angeles, CA, USA) was used to calculate the required sample size. Using data from a previous study, the effect size in our sample size calculation was found to be 0.67 [15]. Based on a power of 80% and a 5% level of significance, the total sample size required was calculated as 74. We evaluated the patients who underwent tracheostomy procedures in a 24-bed intensive care unit between 1 June 2020 and 1 June 2022. Patients under 18 years of age, those tracheostomized in an external center, those admitted to the intensive care unit with mandatory tracheostomy following surgery, and pregnant patients were excluded (Figure 1). Data of the patients who underwent tracheostomy were recorded by an anesthesiologist who is not part of the management team for the patient. Records were started on the day of the procedure, and pre-procedure data (including demographic data, reason for admission, duration of intubation, extubation attempts) were recorded. The included patients were followed up, and the data were recorded.

Intensivists performed percutaneous dilatational tracheostomies at the bedside in the ICUs, whereas Ear–Nose–Throat (ENT) surgeons performed surgical tracheostomies due to difficult anatomy or other predicted risks in the operating theater. Patients were transferred to the operating theater for open tracheostomy. All percutaneous tracheostomies were performed with the forceps dilatation technique without the use of cervical ultrasound or bronchoscopic examination. In the Forseps Dilatation Technique, a reusable set with a special forceps is used. This forceps has been modified from the Howard Kelly forceps by Griggs and colleagues. The tip of the forceps has a channel through which the guide wire can pass. The insertion of the guide wire is similar to the Ciaglia method. The guide wire is passed through the hole at the tip of the forceps, and the subcutaneous tissues and trachea are dilated by opening the forceps in one or two stages. When the stoma reaches a size that can accommodate the cannula, the cannula is inserted into the trachea through the stoma and secured. The patients who met the study criteria were included in the study on the day of tracheostomy. After recording the data prior to tracheostomy, the patients were followed up daily.

All patients included in the study were sedated using our intensive care unit’s standard sedation protocol. Patients who were started on sedation with intravenous propofol infusion received remifentanil infusion when analgesia was needed. Propofol was infused at a dose of 25–75 mcg/kg/min and increased to 5 mcg/kg/min q5–10 min. If analgesia was needed, remifentanil was added with the concentration of 50 mcg/mL. Remifentanil Remifentanil was infused at a dose of 0.1–0.15 mcg/kg/min and increased to 0.025 mcg/kg/min q5 min. We aimed a sedative score of 3 to 4 on the Ramsay sedation scale. 

### 2.3. Data Collection

The patients who met the study criteria were included in the study on the day of tracheostomy. The data prior to tracheostomy including gender, age, comorbidities, APACHE-II score, admission diagnosis, duration of intubation, and extubation attempts were recorded. Data of interest included demographic and past medical history from the day of hospital admission. The following demographic information was collected: age in years, gender, history of hypertension, diabetes mellitus, chronic respiratory disease (defined as chronic obstructive pulmonary disease or asthma), chronic renal disease (defined as estimated glomerular filtration rate < 30 mL/min/1.73 m^2^), chronic heart disease (defined as a history of current or previous cardiac dysfunction), cancer or hematological malignancy, and neurologic disease (any disease of the brain, spinal cord, autonomic nervous system disorder, seizure disorder, movement disorder, migraine, central neuropathy, neuropsychiatric illness).

Sedation requirements, duration of ICU and hospital stay, complications, 90-day mortality, ICU mortality, time to tracheostomy, tracheostomy technique, complications, cannulation status and need for ventilator support at discharge were recorded. We also recorded the number of sedation-free days within 30 days after the tracheostomy.

### 2.4. Definitions

Tracheostomies performed within the first 14 days after endotracheal intubation were classified as early tracheostomy, and those performed at ≥15 days were classified as late. This time was defined as the time from endotracheal intubation to tracheostomy. We defined a sedative day as a day from 7 a.m. to the next 7 a.m. with a cumulative sedative duration more than 6 h. The sedation days in the first 30 days after tracheostomy was subtracted from 30 to determine the number of sedation-free days. Days with sedation of less than 6 h in a day were considered sedation-free days. 

Early complications after tracheostomy were defined as those occurring within the first 7 days. Early complications included bleeding, hypoxia, hypercarbia, emphysema, pneumothorax, and stoma infection. Late complications included stenosis, tracheoinnominate artery fistula, and tracheoesophageal fistula formation. ICU discharge was recorded as the patient’s clinical condition on the last day in the ICU. Discharge to the ward, irrespective of death, ventilator dependency, and decannulation status was recorded.

### 2.5. Statistical Analysis

Statistical analysis was performed using SPSS version 23.0 (IBM Corporation, Armonk, NY, USA). Continuous variables were expressed as mean ± standard deviation (SD), and categorical variables were reported as numbers and frequencies. Normality was assessed using the Shapiro–Wilk test. Quantitative data between the groups were compared using the independent samples *t*-test or Mann–Whitney U test according to the normality of the data. Pearson’s chi-square or Fisher’s exact test was used to compare categorical data between the groups. Statistical significance was accepted as *p* < 0.05.

## 3. Results

In this study, a total of 99 patients underwent tracheostomy, whether percutaneous or surgical, during the same enrollment period (Table 1). We excluded six patients for the incomplete pre-procedure data, and another six patients were excluded from the study due to being transferred to other institutions to increase bed capacity for COVID-ARDS patients during the pandemic. In total, 87 patients were included in the study with tracheostomy performed in the first 14 days (early) for 33 patients and after the 15th day (late) for 54 patients.

The mean age was 66.6 ± 11.7 years in the ET group and 68.5 ± 19.69 years in the LT group, which was significantly higher in the LT group (*p* = 0.049). The sex distribution was not significantly different (*p* = 0.234) between the ET (22 (66.7%) males and 11 (33.3%) females) and the LT 29 (53.7%) males and 25 (46.3%) females) groups. The comorbid diseases consisted of none (*n* = 7, 21.2%), cardiovascular (*n* = 11, 33.3%), hypertension (*n* = 18, 54.5%), and diabetes mellitus (*n* = 3, 9.1%) in the ET group, while they consisted of none (*n* = 1, 1.9%), hypertension (*n* = 37, 68.5%), and diabetes mellitus (*n* = 19, 35.2%) in the LT group. In the early group, 78.8% of patients had at least one comorbid disease, while this rate was 98.1% in the LT group, which was statistically significantly higher.

### 3.1. Extubation Attempts and Weaning Failure 

Thirty-nine patients were ultimately extubated and re-intubated before tracheostomy, while forty-eight were never extubated before tracheostomy. Among those who were extubated, 11 (33.3%) underwent early tracheostomy, while 28 (51.9%) underwent late tracheostomy. Among those never extubated from the endotracheal tube before tracheostomy, 22 (66.7%) underwent early tracheostomy, while 26 (48.1%) underwent late tracheostomy (*p* = 0.092). All the included 87 patients underwent tracheostomy.

### 3.2. Tracheostomy Characteristics

Percutaneous tracheostomy was performed in 59 patients, while the surgical technique was performed in 28 patients. The duration of intubation until tracheostomy was 11.12 ± 3.91 days in the early group and 26.94 ± 11.32 days in the late group, and the latter was significantly longer. The complications that occurred in the early and late periods after tracheostomy were separately analyzed in terms of timing and technique. No significant differences among the groups occurred in terms of adverse events such as bleeding, hypoxia, tracheoesophageal fistula, pneumonia, and stenosis (Table 2). When comparing the complications according to the timing of tracheostomy, no statistically significant difference was found (*p* > 0.05, Table 2). When complications were evaluated according to the tracheostomy technique, it was observed that more complications occurred in patients who underwent tracheostomy surgically, but there was no statistically significant difference between the techniques (*p* > 0.05, Table 2). In our study, tracheoesophageal fistula developed in a total of five patients with four patients in the late tracheostomy group and one patient in the early tracheostomy group.

### 3.3. ICU Mortality, Length of Stay, Sedation-Free Days and Discharge Status

In the 90-day mortality follow-up after tracheostomy, 57.5% of patients died, while 42.5% were alive. When evaluated in terms of the timing of tracheostomy, no significant difference was found (*p* = 0.989). In terms of the relationship between gender and the 3-month mortality after tracheostomy, it was observed that more females died, but there was no significant difference between the genders (*p* = 0.425). The mortality was similar in the groups, while the duration of ICU stay was significantly longer in the cases with late tracheostomy. In the study, the mean APACHE-II score was 17.11 ± 6.29. It was 16.33 ± 7.37 was in the ET group and 17.59 ± 5.54 in the LT group with no significant difference between the groups. 

When comparing the sedation-free days within 30 days after tracheostomy for patients requiring sedation on the day of tracheostomy, there were more sedation-free days in patients who underwent tracheostomy later, but there was no statistically significant difference between the groups (*p* = 0.303, Table 3). Successful decannulation was defined as successful tracheostoma removal within three months whether in the ICU or not. At the end of the study, 18 patients were ventilator-free but tracheostomized, and five patients were decannulated (Table 3). We did not observe a positive influence of ET on decannulation.

## 4. Discussion

Within our particular patient group, we found that performing a tracheostomy on the ≤14th day of intubation had no effect on sedation requirements, ventilator weaning, decannulation, or mortality. However, tracheostomy performed ≥14 days increases the intensive care unit. It was observed that post-tracheostomy complications were similar in both surgical and percutaneous technique. We thought that the similar complication rates might be due to the fact that the percutaneous technique was not performed under bronchoscopy guidance. 

Hypertension, diabetes, and cardiac and neurological diseases are the most common comorbidities in ICUs. Among the most common reasons for admission to the ICU are neurological causes, respiratory failure, cardiac problems, and trauma. It has been reported that the most common reason for admission in patients requiring tracheostomy due to prolonged intubation is neurological causes [16]. In our study, comorbid diseases were more common in the late tracheostomy group. If we also consider that the patients in the late tracheostomy group were at a more advanced age, we can conclude that specialists may delay tracheostomy due to a lower life expectancy.

In ICUs, tracheostomy in patients with prolonged intubation aims to reduce the sedation requirements, facilitate easier airway clearance, and ultimately decrease the need for mechanical ventilation [13,14]. In a survey conducted with 429 specialists from 59 different countries, it was shown that tracheostomy was most commonly performed due to prolonged mechanical ventilation and difficulty in weaning (77.9%), and the most common timing for the procedure was between days 7 and 15 (54.4%) [14]. Tracheostomy was almost always preferred due to prolonged intubation in our study. The optimal timing for tracheostomy to ensure it has a positive impact on survival, hospital stay, or intubation complication in the intensive care setting is a subject of debate. While the definition of an early tracheostomy remains controversial, there is greater consensus on classifying tracheostomies performed at ≥14 days as late-term interventions [17]. The term ‘early tracheostomy’ is used differently in the literature; we defined the tracheostomies performed at ≤14 days as early. We observed that tracheostomy was frequently performed in the late period in our ICUs. The reasons for this may include the advanced age of patients, lower expected benefits from tracheostomy, and the hesitancy of patients’ relatives to give consent for the tracheostomy procedure, citing the advanced age of the patients. We observed that particularly in elderly patients admitted for neurological reasons and with a high clinical frailty prior to admission, the families struggled to understand the benefits of a tracheostomy. 

The significant heterogeneity in patient, disease, and clinician approaches makes it difficult to determine the appropriate timing of tracheostomy in cases of prolonged intubation. This lack of standardization hinders the observation of the benefits of early tracheostomy. In a prospective, randomized study involving 120 patients, it was reported that performing tracheostomy on the 2nd day of intubation reduced the 30-day mortality, decreased the risk of developing pneumonia, and shortened the duration of stay in the ICU by half compared to a tracheostomy performed between the 14th and 16th days [18]. In a retrospective study of 153 COVID patients who underwent tracheostomy on or after the 15th day of intubation, early tracheostomy was found to be associated with shorter ICU stay but unrelated to mortality [19]. A review conducted in 5106 patients with traumatic brain injury (ET group: 2509, LT group: 2597) reported that early tracheostomy could provide a shorter duration of mechanical ventilation, a shorter length of ICU and hospital stay and a decrease in adverse events caused by prolonged intubation [11]. On the other hand, a meta-analysis including 17,346 patients diagnosed with stroke showed that the timing of tracheostomy was not associated with the mortality, ICU/hospital length of stay, or neurological outcomes [20]. In another retrospective study where they analyzed the effects of timing in unspecified ICU patients, they divided the tracheostomy timing into three categories (early, ≤4 days; intermediate, 5–9 days; late, ≥10 days). They found that early tracheostomy (≤4 days) did not affect weaning success but could be beneficial in reducing the duration of mechanical ventilation and possible complications of intubation [1]. In a retrospective cohort study involving 1884 general intensive care unit patients, the patients were divided into subgroups based on their admission diagnoses, and tracheostomies performed within 7 days, between 7 and 14 days, and after 14 days were examined. That study found that delayed tracheostomy was associated with prolonged ICU stay, increased sedation requirements, and prolonged mechanical ventilation duration [21].

Although there are studies showing that early tracheostomy reduces mortality, an observational study conducted in stroke patients in 2023 showed that the timing of tracheostomy was unrelated to mortality or length of stay [20]. In their study of 153 COVID patients, Battaglini et al. showed that tracheostomies performed before the 15th day of intubation reduced the length of stay but were unrelated to mortality [19]. In our study, we found no relationship between mortality and the timing of tracheostomy. However, we observed that the length of stay was significantly longer in patients who underwent delayed tracheostomy. In the late tracheostomy group, history of neurological disease was significantly high. This could be attributed to clinicians’ tendency to postpone tracheostomy in these patients with poor prognosis.

One of the foremost expectations from tracheostomy is to reduce the number of sedation days. However, there are studies indicating that the timing of tracheostomy does not affect the sedation requirement, and there are also studies indicating that there are more sedation-free days when tracheostomy is performed early [22,23,24]. In our study, days with a sedation intake of less than 6 h were considered as sedation-free days; we observed the patients who needed sedation between the day of tracheostomy and 30 days following tracheostomy and found that there were no more sedation-free days in the patients who underwent early tracheostomy. 

Percutaneous dilatational tracheostomy has been shown to have several advantages compared to surgical tracheostomy, including a smaller skin incision, less tissue damage, reduced bleeding, a lower risk of stoma infection, a quicker opening, bedside applicability, a lower cost due to the absence of the need for personnel, equipment, and an operating room, and the potential for being performed by non-surgical medical professionals [8]. The choice of technique can vary depending on patient-related factors or the clinician’s preference and experience. While surgical tracheostomy is mostly preferred in France [8], in Germany, 86% of tracheostomies are performed at the bedside by anesthetists using the percutaneous method [25]. In addition, the frequency of bronchoscopy use to prevent iatrogenic events in the percutaneous technique varies significantly depending on experience and hospital resources. Despite its disadvantages, such as potentially disrupting ventilation and causing CO2 retention, increasing costs, and prolonging the procedure, bronchoscopy is recommended to prevent injuries to the posterior tracheal wall [8]. In our study, the percutaneous tracheostomy method was more frequently performed without broncoscopy for a total of 59 patients (67.8%).

The higher rate of complications in the surgical method is thought to be due to the redirection of complex patients and those anticipated to be challenging cases toward the surgical approach [9]. Although this situation implies that the results of retrospective studies may not be conclusive, there are randomized controlled prospective studies supporting the results of our study [10]. In the current study, complications were more frequently observed in both late and surgically opened tracheostomies, although they were not statistically significant. When compared to the literature, our observed complication rate, which appears to be higher, may related to the referral of complex patients to our hospital.

With regular respiratory rehabilitation, patients can be successfully decannulated after days without the need for mechanical ventilation, leading to a potential reduction in the length of the hospital stay. In the current study, at the time of discharge, five patients were decannulated, while thirteen patients were cannulated but free from the ventilator. When evaluated in terms of weaning success, we did not find superiority in the tracheostomies performed early. Since there is no consensus on which day tracheostomy should be performed for superior prognosis, the clinician’s decision should be based on individual patient factors, the risk–benefit ratio, the expected prognosis, and the expectations of the patient and their family. 

Our current study has several limitations that need to be acknowledged. First, the underlying reasons for the tracheostomy timing could not be clearly determined. However, the aim of the study was to collect detailed data on intensive care patients who underwent tracheostomy after failed extubation. In order to obtain more information, there is a need for prospectively randomized studies designed in similar patient groups. Second, the variability in tracheostomy timing among physicians could not be excluded as a contributing factor. Third, the majority of patients were discharged without being decannulated. The relatively high 90-day mortality may be attributed to this. This suggests that care was provided to patients with relatively low survival rates. At this point, the results of our study may be debatable, but a prospective study design incorporating appropriate follow-up observations would be useful.

## 5. Conclusions

As a conclusion, early tracheostomy does not have a positive effect on mortality, sedation duration, or decannulation. However, the intensive care unit stay was significantly longer with late tracheostomies. Although complications are less common with percutaneous tracheostomy, it may not be superior to surgical procedures. Performing percutaneous tracheostomy without bronchoscopy guidance may decrease the frequency of complications. 

The decision for tracheostomy varies depending on the patient’s clinical condition, life expectancy, and the clinician’s judgment. Further prospective studies are needed that focus on early tracheostomy in specified patients who cannot be weaned successfully.

## Figures and Tables

**Table 1:** Overall characteristics of 87 tracheostomized patients.

	Early Tracheostomy *n* = 33	Late Tracheostomy *n* = 54	*p* Value
Sex (Female)	11 (33.3%)	25 (46.3%)	0.234
Respiratory-related intubation	10 (30.3%)	29 (53.7%)	0.116
Comorbidities			
None	7 (21.2%)	1 (1.9%)	0.004 *
Hypertension	18 (54.5%)	37 (68.5%)	0.190
Diabetes mellitus	3 (9.1%)	19 (35.2%)	0.007 *
Coronary artery disease	11 (33.3%)	13 (24.1%)	0.348
Chronic respiratory disease	4 (12.1%)	5 (9.3%)	0.725
Chronic renal disease	2 (6.1%)	5 (9.3%)	0.705
Neurologic disease	7 (21.2%)	28 (51.9%)	0.007 *
Malignancy	2 (6.1%)	4 (7.4%)	>0.99
Admission diagnosis			
Respiratory failure	11 (33.3%)	31 (57.4%)	0.029 *
Circulatory failure	1 (3%)	2 (3.7%)	>0.99
Neurology	3 (9.1%)	6 (11.1%)	>0.99
Multi-trauma	10 (30.3%)	7 (13%)	0.048 *
Post-CPR	7 (21.2%)	7 (13%)	0.31
Age	59.91 ± 19.04	68.5 ± 19.69	0.049

* *p* < 0.01. Continuous data are presented as mean ± standard deviation, categorical data by frequencies and percentages. CPR: cardiopulmonary resuscitation.

**Table 2:** Tracheostomy characteristics.

	Early Tracheostomy *n* = 33	Late Tracheostomy *n* = 54	*p* Value
Time to tracheostomy (days) mean ± SD	11.12 ± 3.91	26.94 ± 11.32	0.001 *
Technique, *n* (%)			
Percutaneous	24 (72.7%)	35 (64.8%)	0.443
Surgical	9 (27.3%)	19 (35.2%)	
Complications, *n* (%)			
None	29	35	0.057
Hemorrhage	0	6	
Hypoxia	0	2	
Emphysema	1	0	
Pneumonia	1	6	
Tracheoesophageal fistula	1	4	
Tracheal stenosis	1	1	

* *p* < 0.01 Continuous data are presented as mean ± standard deviation, categorical data are presented by frequencies and percentages.

**Table 3:** Primary and secondary endpoints.

	Early Tracheostomy *n* = 33	Late Tracheostomy *n* = 54	*p* Value
ICU mortality	24 (72.7%)	44 (81.5%)	0.339
90-days mortality	19 (57.6%)	31 (57.4%)	0.988
ICU LOS (days)	48.36 ± 26.35	66.96 ± 36.11	0.001 *
Sedation-free days	18.26 ± 10.75	20.89 ± 10.15	0.303
Discharge status			
Tracheostomized	30 (90.9%)	52 (96.2%)	0.363
Ventilator-free	5 (15.1%)	8 (14.8%)	0.522
Decannulated	3 (9.1%)	2 (3.7%)	0.363

* *p* < 0.01 Continuous data are presented as mean ± standard deviation, categorical data are presented by frequencies and percentages. ICU: intensive care unit, LOS: length of stay.

## Data Availability

The data presented in this study are available on request from the corresponding author. The data are not publicly available because of patient privacy and data protection regulations.
